# Community-based intervention to promote breast cancer awareness and screening: The Korean experience

**DOI:** 10.1186/1471-2458-11-468

**Published:** 2011-06-14

**Authors:** Keeho Park, Woi Hyun Hong, Su Yeon Kye, Euichul Jung, Myung-hyun Kim, Hyeong Geun Park

**Affiliations:** 1 Cancer Information and Education Branch, National Cancer Center, Goyang, Republic of Korea; 2 School of Nursing Science, Kyungnam University, Masan, Republic of Korea; 3 School of Journalism, Media and Advertising, Sangji University, Wonju, Republic of Korea

## Abstract

**Background:**

There are many differences in culture, community identity, community participation, and ownership between communities in Western and Asian countries; thus, it is difficult to adopt the results of community intervention studies from Western countries. In this study, we conducted a multicity, multicomponent community intervention trial to correct breast cancer myths and promote screening mammography for women living in an urban community in Korea.

**Methods:**

A 6-month, 2-city community intervention trial was conducted. In the intervention city, 480 women were surveyed at baseline and 7 months later to evaluate the effects of the intervention program. Strategies implemented in the intervention city included community outreach and clinic and pharmacy-based in-reach strategies.

**Results:**

This study showed a 20.4-percentage-point decrease in myths about the link between cancer and breast size, a 19.2-percentage-point decrease in myths concerning mammography costs, and a 14.1-percentage-point increase in intention to undergo screening mammography. We also saw a 23.4-percentage-point increase in the proportion of women at the action stage of the transtheoretical model in the intervention city. In the comparison city, smaller decreases and increases were observed.

**Conclusions:**

Our study showed the value of an intervention study aimed at reducing belief in breast cancer myths in an urban community in Korea. The invention also made women more likely to undergo mammography in future.

## Background

Cancer has been the leading cause of death in the Republic of Korea since 1983. Approximately 140,000 people develop cancer annually with 65,000 annual fatalities. Cancer control is an important issue because of the country's rapidly aging society and the subsequent increased burden of cancer. The National Cancer Screening Program (NCSP) offers Medical Aid users and those National Health Insurance (NHI) beneficiaries who fall within the lower 50 percent income bracket free screening for 5 common cancers--cancer of the stomach, liver, colorectum, breast, and cervix uteri. For NHI beneficiaries in the upper 50 percent income bracket, the maximum cost of a mammogram is 6 dollars. The NCSP recommends biennial mammograms for women over 40 years of age. However, only 49.5% of women act in accordance with these guidelines.

Although mass media health communication strategies can effectively promote health education, and influence health awareness, decisions, practices, and care [1], interpersonal communication channels are regarded as highly influential to persuade people to change health-related behaviors [2]. In addition, because it is difficult for one-way mass communication strategies driven by the central government to challenge strong beliefs or shifting attitudes [3], the Second Term (2006-2015) of the 10-year Comprehensive Plan for Cancer Control in Korea focuses on the idea of community-based health communication to promote cancer screening.

There are more than 250 Public Health Centers (PHCs) in Korea. These organizations are part of the National Health System and are operated by local governments to prevent and control diseases or tackle hygiene problems at the county/district level. Despite the presence of this strong community-based health service network, most PHCs have struggled to use theory or evidence-based approaches to promote cancer screening. However, with the new emphasis on community-based health communication in the Second Term (2006-2015) of the 10-year Comprehensive Plan for Cancer Control, these PHCs are now looking at ways to gather evidence. One difficulty they face is that most of the research on community campaigns for promoting cancer screening has been conducted in Western countries or in non-Asian populations. As there are many differences in culture, community identities, community participation, and ownership between communities in Western and Asian countries, it is not possible to directly adopt the results of studies from Western countries.

In this study, we conducted a community-based intervention study to correct myths related to breast cancer and promote screening mammography for women living in an urban community in Korea, Gunpo. The theoretical framework for the community-based interventions included the PRECEDE/PROCEED model for planning [4] as well as the health belief model (HBM) [5,6], Transtheoretical model (TTM) [7] and social marketing [8]. The PRECEDE/PROCEED model is a popular road map for health promotion programs. The model views health behavior as influenced by both individual and environmental forces, and its 2 parts comprise an educational (PRECEDE) and an ecological diagnosis (PROCEED). Using the model, we conducted social, epidemiological, behavioral, environmental, educational, ecological, administrative, and policy assessment with the help of the Gunpo PHC. We also assessed the factors that predict why people will take action to screen for breast cancer using the HBM; these include susceptibility, seriousness, benefits and barriers, cues to action, and self-efficacy. The stage construct is important because it represents a temporal dimension. In the past, behavior change often was construed as a discrete event. The TTM posits change as a process that unfolds over time, with progress through a series of stages, although frequently not in a linear manner. To stage the mammography, we identified the past history of breast cancer screening, recent breast cancer screening, and future breast cancer screening intention.

We chose the social marketing strategy approach to promoting health behavior. We did not merely try to inform people or persuade them to change their behavior, but attempted to sell our services as products. We analyzed the target population using segmentation by age and TTM stage of change, and developed intervention activities according to results of the analysis. Because breast screening with the NCSP is free for Korean women aged 40 years and over and because community health education is also free, we regarded "place" and "promotion" as the main elements of the marketing mix (Product, Price, Place, and Promotion) needing to be addressed. Therefore, we adopted an outreach education program and direct mailing as campaign activities.

The Gunpo Cancer Screening Project (GCSP) aimed to identify barriers to breast cancer screening and address these barriers in a multicomponent program designed to improve beliefs, attitudes, and screening behaviors for breast cancer.

## Methods

### Survey

We used the HBM and the TTM to develop the questionnaire for our quantitative research into health behaviors. A cross-sectional face-to-face survey using structured questionnaires was conducted with randomly selected sample of 503 women aged 30-69 years who were permanent residents in the intervention city. Self-administered questionnaires were used to collect sociodemographic data. Survey was conducted to assess predisposing factors such as perceived risk of breast cancer, knowledge on breast cancer and breast screening, perceived severity of breast cancer, awareness of breast cancer screening, perceived barriers to breast cancer screening, satisfaction with recent breast cancer screening, and self-efficacy, and reinforcing factors which are recommendations from physician or pharmacist, past history of cancer and other chronic diseases, past history of benign breast diseases, and family history of breast cancer, and finally enabling factors, for example, perceived cues to action and factors affecting hospital choice for breast cancer screening.

This study was approved by the Institutional Review Board of the National Cancer Center.

### Interviews

Health workers from the health promotion and cancer control program department in the PHC were interviewed in their role as key administrative and policy informants. We explored their current health promotion activities including their cancer control program, policy, enabling factors for the regional cancer control program, perceived barriers to the regional cancer control program, and characteristics of the PHC organization and those staff conducting health promotion. We conducted eleven focus group interviews; questions were based on stages of mammography adoption using the TTM and the HBM constructs. The results of both qualitative and quantitative research were used to develop educational materials for small group education sessions. Since this study represents only the quantitative data, we do not report the findings of qualitative research.

### Message

The main message of the community campaign was that screening mammography can detect masses which are not palpable. We brainstormed messages that matched our communication campaign goal of correcting myths about breast cancer and screening mammography. These were pretested and we revised the message with the members of the Community Advisory Committee. Posters were then drawn up.

### Target area

Gunpo was selected for the intervention for several reasons. First, the screening rate for breast cancer was about average for Korean PHCs. Second, physical accessibility to clinics or hospitals was not a significant barrier for cancer screening. Third, its geographic and demographic characteristics allowed for generalizability for urban Korea, which is where approximately half of all Koreans live. Fourth, Gunpo is neither too large nor too small to implement a community-based intervention trial with a limited budget.

With a land area of 36.38 km^2^, Gunpo is located in the metropolitan area near Seoul. As of December 2007, it had a total population of 275,351 (men 137,718; women 137,633). Gwang Myeong was selected as a comparison region. We selected it because it has similar geographic and sociodemographic characteristics and because it is far enough away from the intervention city to ensure the presence of a buffer zone should the intervention "contaminate" beyond the intervention city's boundaries. Gwang Myeong is also located in the metropolitan area around Seoul with a total population of 311,700 (men 155,407; women 156,293) and a land area of 38.50 km^2^.

### Interventions

To develop effective interventions, we used results from the formative research, additional focus interviews, and input from the Community Advisory Committee. Interventions implemented in Gunpo over 6 months included: (a) posters on apartment billboards; (b) posters in clinic waiting rooms; (c) posters on pharmacy walls; (d) leaflets distributed at street events; (e) direct mailing to promote breast cancer screening; (f) street promotion; (g) outbound calls to women who signed application forms at the street promotions, monthly neighborhood meetings, or small group educational sessions; (h) small group educational sessions; and (i) a blog on breast cancer screening.

We obtained informed consent from all the participants who were contacted by the study team.

### Evaluation

We conducted pre-intervention baseline surveys in June 2008. This cross-sectional face-to-face survey using structured questionnaires was conducted with a random sample of 240 women aged 30-69 years from the intervention city and another 240 from the comparison city. Phase 2 began at the conclusion of the intervention delivery, approximately 7 months after the baseline survey was concluded, and involved a follow-up survey of women. The post-intervention survey was conducted with an independent sample of 240 women from each city.

For campaign and non-campaign exposure, we included items for measuring possible non-GCSP campaign activities in the questionnaire. This was done in order to control the confounding effect of those extrinsic activities, which could act as "noise," and make it more difficult to assess the effectiveness of the main GCSP campaign. For dose-exposure questions, participants were asked 3 questions with a 7-point Likert-type scale for each communication activity. The questions asked: (a) if they had been exposed to the campaign activity for breast cancer screening, ranging from "never seen" to "seen very frequently;" (b) if they had been able to see the activity in detail, ranging from "never could see it in detail" to "could see very detailed information;" and (c) if it was easy to remember the activity, ranging from "could not remember the activity at all" to "could remember the activity very clearly." Overall media exposure was measured as the average of each respondent's scores across this 3-item, 7-point Likert-type scale. There was no prompting for these questions. The instrument for measuring campaign exposure modified the framework used by a top advertising agency in Korea.

### Mammography myths and intention toward

Respondents responded to the following 7 statements or questions: (a) most lumps suspicious of breast cancer are painful; (b) women whose breast size is bigger are more likely to get breast cancer; (c) the best time to get a mammogram is when there are breast symptoms; (d) we do not need to get a mammogram when no abnormal signs or symptoms are found in breast self examination; (e) mammograms are expensive; (f) women older than 60 do not need a mammogram; (g) do you intend to get a mammogram within 2 years? These questions were based upon formative research conducted in the first phase. The myth assessment items were included in the questionnaire because they had received lower scores in the formative quantitative survey on breast cancer awareness, health status, lifestyle, health consciousness, health behavior, knowledge on breast cancer, perceived barriers toward cancer screening, and self efficacy.

Stage of mammography adoption by TTM was assessed using women's responses to the questionnaire on their intention of obtaining a mammogram in the coming 2 years, asking whether they had been screened in the previous 2 years, and looking at their reported history of mammography use in the previous 2 years[7].

### Analysis

The basic characteristics on demography and past history of mammography (see Table 1) were calculated separately at baseline and follow-up for both cities. Differences between cities were assessed using χ^2 ^tests. We used t-tests to compare campaign recall scores between baseline and follow-up for each city. To assess whether the intervention was related to a beneficial change in myths with respect to breast cancer and screening mammography, χ2 tests were used. The intervention's myth busting effect was assessed using unadjusted logistic regression models. Model factors included TIME (baseline/follow-up), CITY (intervention/comparison), and a TIME by CITY interaction term. This interaction term tested the differential effect of the intervention. To determine which intervention activity was related to correct answers (correct understanding) for 6 questions related to screening mammography, a series of logistic models was fitted. We developed models using forward stepwise logistic regression for the following: subjects' characteristics; CITY, TIME, CITY by TIME interaction; all of the intervention activities listed in Table 2; other possible non-campaign activities; and all of the 2- and 3-way interactions of these variables with CITY, TIME, and CITY by TIME. Odds ratios and 95% confidence intervals were produced in the final models. The activities were judged to be related to the outcomes if there were significant 3-way interactions between the activities, CITY and TIME.

**Table 1:** Demographic characteristics by city and time

Variable	Intervention		Comparison	
	
	Baseline (n = 240) n (%)	Follow-Up (n = 240) n (%)	Baseline (n = 240) n (%)	Follow-Up (n = 240) n (%)
Age (yr)				
30-39	93 (38.8)	93 (38.8)	90 (37.5)	90 (37.5)
40-49	83 (34.6)	83 (34.6)	78 (32.5)	78 (32.5)
50-59	38 (15.8)	38 (15.8)	43 (17.9)	43 (17.9)
60-69	26 (10.8)	26 (10.8)	29 (12.1)	29 (12.1)
Currently married^*a *^	225 (93.8)	239 (99.6)	219 (91.2)	227 (94.6)
Education ≤ 12 yr^*b *^	139 (57.9)	135 (56.2)	172 (71.7)	173 (72.1)
Annual household income < $20000	27 (11.2)	7 (2.9)	15 (6.2)	16 (6.7)
Employed^*c *^	92 (38.3)	30 (12.5)	66 (27.5)	120 (50.0)
History of mammography (yes)^*d *^	147 (61.2)	176 (73.3)	122 (50.8)	128 (53.3)

^*a*^*P *< 0.05 at follow-up between cities.

^b c d ^*P *< 0.05 at baseline and follow-up between cities.

**Table 2:** Recall scores for community activities by city and time

Exposure	Intervention			Comparison		
	
	Baseline (n = 240) Mean (SD)	Follow-Up (n = 240) Mean (SD)	p value	Baseline (n = 240) Mean (SD)	Follow-Up (n = 240) Mean (SD)	p value
Posters on apartment billboards	2.81 (1.72)	3.54 (1.34)	< 0.001	3.07 (1.40)	3.40 (1.75)	0.023
Posters in clinic waiting rooms	3.74 (1.87)	3.97 (1.32)	0.128	4.13 (1.77)	3.85 (1.76)	0.084
Leaflets	2.55 (1.88)	3.71 (1.32)	< 0.001	2.56 (1.32)	3.28 (1.87)	< 0.001
Direct mail	2.50 (1.77)	3.53 (1.39)	< 0.001	2.77 (1.43)	3.06 (1.86)	0.054
Street promotion	1.71 (1.08)	2.68 (1.11)	< 0.001	2.33 (1.14)	2.92 (2.17)	< 0.001
Website (National Cancer Information Center)	1.34 (0.86)	2.48 (1.35)	< 0.001	1.61 (1.01)	1.71 (1.45)	0.382
Outbound call	1.38 (0.76)	2.65 (1.39)	< 0.001	1.70 (1.00)	2.11 (1.65)	0.001
Small group education by GCSP	1.81 (1.22)	3.07 (1.29)	< 0.001	2.30 (1.40)	2.70 (1.95)	0.010

SD, standard deviation; GCSP, Gunpo Cancer Screening Project.

## Results

Sociodemographic variables and past history of mammography of the sample in the pre- and post-intervention surveys are shown in Table 1 by both city and time period. There was no significant age difference between study samples in the intervention and control cities. However, there were more married women in the intervention city at the follow-up, and women in the comparison city had lower levels of educated at both baseline and follow-up. While there were more employed women in the intervention city at baseline, more employed women were in the sample of the comparison city at the follow-up. More women in the intervention city had a mammogram history. At follow-up, recall scores were significantly increased in the intervention city for all campaign activities except for seeing posters in clinic waiting rooms (Table 2). The baseline exposure scores for the intervention city were lower than scores for the comparison city on every activity. Scores for 5 of the 8 activities were significantly increased in the intervention city at follow-up. In addition, the differences in average recall scores between baseline and follow-up were greater in intervention city for all campaign activities.

In terms of changes to beliefs in breast cancer myths, there were significant decreases in the proportion of women who believed that bigger breast size raised the likelihood of breast cancer, and in the proportion of women who thought that mammograms were expensive in the intervention city (Table 3). Significant change was also observed in the proportion of those who intended to have screening mammography. However, significant changes in the opposite direction were observed for 4 myths in the comparison city ("most lumps suspicious of breast cancer are painful;" "women whose breast size is bigger are more likely to get breast cancer;" "the best time to get a mammogram is when there are breast symptoms;" "we do not need to get a mammogram when no abnormal signs or symptoms are found in breast self examination"). When we assessed whether there were differential effects of the intervention on the beneficial changes in myths using unadjusted logistic regression models including TIME, CITY, and a TIME by CITY interaction term, significant results were observed for 3 myths ("most lumps suspicious of breast cancer are painful;" "women whose breast size is bigger are more likely to get breast cancer;" "the best time to get a mammogram is when there are breast symptoms"). While no beneficial change was observed in the comparison for these 3 myths, the intervention city showed a significantly decreased level of belief of those myths. For example, people in the intervention city were 3.87 times more likely to have decreased level of belief of those myths "most lumps suspicious of breast cancer are painful". Results for our comparison of changes in the TTM stage of mammography adoption between intervention and comparison cities are shown in Table 4. The proportions for contemplation and action when added together increased from 77.1% to 91.3% in the intervention city, while there was a small increase from 82.9% to 90.0% in the comparison city.

**Table 3:** Percentage of survey respondents reporting outcomes pre- and post-campaign in intervention and comparison cities

Outcomes	Intervention			Comparison			
	
	Baseline (n = 240) n (%)	Follow-Up (n = 240) n (%)	***P***	Baseline (n = 240) n (%)	Follow-Up (n = 240) n (%)	*P*	OR (CI)*
Myth 1-Most lumps suspicious of breast cancer are painful (No)	164 (68.3)	172 (71.7)	0.486	189 (78.8)	127 (52.9)	< 0.001	3.87 (2.21-6.76)
Myth 2-Women whose breast size is bigger are more likely to get breast cancer (No)	108 (45.0)	157 (65.4)	< 0.001	154 (64.2)	116 (48.3)	0.001	4.43 (2.63-7.44)
Myth 3-The best time to get a mammogram is when there are breast symptoms (No)	184 (76.7)	194 (80.8)	0.315	212 (88.3)	165 (68.8)	< 0.001	4.42 (2.31-8.46)
Myth 4-We do not need to get mammogram when no abnormal signs or symptoms are found in breast self examination (No)	214 (89.2)	192 (80.0)	0.008	192 (80.0)	170 (70.8)	0.026	0.80 (0.41-1.56)
Myth 5-Mammograms are expensive (No)	67 (27.9)	113 (47.1)	< 0.001	53 (22.1)	90 (37.5)	< 0.001	1.09 (0.63-1.89)
Myth 6-Women older than 60 do not need a mammogram (No)	194 (80.8)	192 (80.0)	0.908	208 (86.7)	199 (82.9)	0.309	1.27 (0.65-2.49)
Do you intend to get a mammogram within 2 years?	185 (77.1)	219 (91.2)	< 0.001	199 (82.9)	216 (90.0)	0.032	1.67 (0.78-3.59)

*City-by-time interaction effects; OR, odds ratio; CI, confidence interval

**Table 4:** Stages of change of mammography adoption in intervention and comparison cities

TTM stages	Intervention			Comparison		
	
	Baseline (n = 240) n (%)	Follow-Up (n = 240) n (%)	*P*	Baseline (n = 240) n (%)	Follow-Up (n = 240) n (%)	*P*
Precontemplation	34 (14.2)	16 (6.7)	< 0.001	35 (14.6)	14 (5.8)	0.006
Relapse	17 (7.0)	3 (1.2)		2 (0.8)	4 (1.7)	
Relapse risk	4 (1.7)	2 (0.8)		4 (1.7)	6 (2.5)	
Contemplation	96 (40.0)	74 (30.8)		92 (38.3)	120 (50.0)	
Action	89 (37.1)	145 (60.5)		107 (44.6)	96 (40.0)	

TTM, Transtheoretical model

In particular, there was profound change in the proportion for the TTM action stage in the intervention city.

We explored whether the GCSP campaign activities were related to correct understanding of the 6 myths, and to intention regarding screening mammography (Table 5 and 6). Possible secular trends and the influence of non-campaign activities were statistically controlled by fitting the multivariate logistic models with the following: characteristics of the subjects; CITY, TIME, CITY by TIME interaction; all of the intervention activities listed in Table 2; other possible non-campaign activities; and all of the 2- and 3-way interactions of these variables with CITY, TIME, and CITY by TIME. When a certain campaign activity in the intervention city has a statistically significant interaction with CITY by TIME, we understand that the activity is associated with the campaign of the intervention city. Posters on apartment billboards were associated with understanding that the myth "most lumps suspicious of breast cancer are painful" is not true. Clinics or pharmacy waiting room posters were associated with recognizing the untruth of "women whose breast size is bigger are more likely to get breast cancer." Street promotions and recommendation by physicians or pharmacists were associated with discrediting the myth that "the best time to get a mammogram is when there are breast symptoms." Street promotions were associated with an end to the idea that "we do not need to get a mammogram when no abnormal signs or symptoms are found in breast self examination." However, no activity was found to be associated with an end to the belief that "mammograms are expensive." Street promotions and personal stories of cancer patients were associated with dispelling the myth that "women who are older than 60 do not need a mammogram."

**Table 5:** Logistic regression models for factors related to change in myths about screening mammography (n = 480)*

	Odds ratios and 95% confidence intervals					
	**Myth 1**	**Myth 2**	**Myth 3**	**Myth 4**	**Myth 5**	**Myth 6**

Age (yr) × Time × City						
30-39				1.0		1.0
40-49				1.67 (0.70-4.00)		1.08 (0.33-3.57)
50-59				0.34 (0.14-0.84)		0.19 (0.05-0.74)
60-69				0.50 (0.18-1.45)		0.13 (0.03-0.53)
Marital status × Time × City (currently married vs. not currently married)	5.17 (1.20-22.25)					
Income × Time × City (≥ 20000$ vs. < 20000$)						33.39 (4.67-238.45)
Employment × Time × City						27.46 (1.97-382.29)
History of mammography × Time × City		2.48 (1.32-4.63)				
TV ads on breast cancer screening × Time × City				0.65 (0.52-0.82)		
Radio ads on breast cancer screening × Time × City			0.71 (0.50-1.00)			
Newspaper article or ad × Time × City	0.63 (0.46-0.88)					0.35 (0.18-0.68)
Posters on apartment billboards × Time × City	2.12 (1.47-3.05)					
Posters in clinic or pharmacy waiting rooms × Time × City		1.40 (1.17-1.68)				
Street promotion × Time × City			2.30 (1.53-3.47)	1.59 (1.15-2.20)		1.59 (1.03-2.47)
Ad on other websites × Time × City			0.48 (0.31-0.72)			
Physician or pharmacist recommendations × Time × City			1.74 (1.29-2.34)			
Personal stories of cancer patients × Time × City						1.78 (1.01-3.16)
Small group education by private hospitals × Time × City	0.64 (0.46-0.89)					0.34 (0.20-0.57)

* Only variables that had a time by city interaction term are shown in the table because of the high number of variables involved in the final model.

**Table 6:** Logistic regression models for factors related to change in intention toward screening mammography (n = 480)

		95% CI for odds ratio	
	**Odds ratio**	**Lower**	**Upper**

Marital status (currently married vs. not)	2.03	1.02	4.02
Household income (≥ 20000$ vs. < 20000$)	2.58	1.39	4.80
History of mammography	4.61	2.86	7.42
Radio ads on breast cancer screening	0.84	0.72	0.98
Posters in clinics or pharmacy waiting rooms	1.33	1.17	1.51
Age (yr) × City			
30-39	1.0		
40-49	0.92	0.47	1.80
50-59	0.41	0.18	0.95
60-69	0.21	0.09	0.48
Outdoor advertising on breast cancer screening in other cities × Time	1.37	1.14	1.64
History of mammography × Time × City	4.92	1.39	17.42
Direct mail × Time × City	1.49	1.00	2.03
Personal stories of cancer patients × Time × City	0.59	0.42	0.84

CI, Confidence interval

In the multivariate logistic regression, marital status, household income, history of mammography, and posters in the waiting rooms of clinics or pharmacies were significantly associated with intention toward screening mammography regardless of the campaign (Table 6). Finally, direct mail was independently associated with intention regarding screening mammography. Conversely, personal stories of cancer patients were inversely associated with intention with regards to screening mammography.

## Discussion

The goal of this study was to explore the effect of a multifaceted community intervention trial on correcting myths related to breast cancer and screening mammography. The study also aimed to increase women's intention toward screening mammography, and improve the TTM stage of adoption for screening mammography for women aged 30 years and older. The effects of the intervention tested in this study included a 20.4-percentage-point decrease in myths about the link between breast size and breast cancer, a 19.2-percentage-point decrease in myths about costs for mammography, and a 14.1-percentage-point increase in intention regarding screening mammography. There was also a 23.4-percentage-point increase in the proportion of women at the action stage of TTM.

To our knowledge, this is the first multi-city community intervention study to apply multicomponent interventions including direct mail conducted in an Asian country. The campaign achieved many of its aims despite a relatively short duration and low budget (14,250 US dollars). Most components relied on volunteers, low-cost media, and participation in community events. In this community intervention study, the combination of community outreach (posters on apartment billboards, street promotions, direct mailing, and educational sessions) and clinic and pharmacy-based in-reach (posters in clinic waiting rooms or pharmacies and physician or pharmacist recommendations) strategies was related to improve in campaign outcomes.

The level of penetration of the intervention was weak to modest. Measurement was complicated by a background effect represented by baseline scores; this was because many agencies and health professionals may promote cancer screening. Also, the possible presence of concurrent communication activities by other projects in a control city may limit the effect of a campaign [9]. Besides, the presence of a concurrent national message could make it difficult for a local campaign to have an additional effect in the intervention city [9]. We could not demonstrate an impact of the intervention on those 4 myths for which there was already a high level of correct understanding. The "ceiling effect" might be playing a role here. On the other hand, there was evidence that GCSP did have effects on myths about breast size and cost for mammography, and on intention with regards to screening mammography. Even though there were no significant change in intervention city for myths on breast cancer symptoms and the best time for mammography, we found differential effects of the intervention on the beneficial changes in belief in myths.

It is strange that there were also increases or decreases in myths in the comparison city. However, it is possible that there is a secular trend toward change in the control city. When such a trend exists, the effect of a campaign may be attenuated [9]. Specifically, a positive trend could be the result of a concurrent national message or some form of non-campaign communication in the control community.

Even though the use of mass media, such as posters or leaflets, can convey simple information and increase knowledge, it can only change behavior if there are facilitating factors [10]. Therefore, we tried to boost interpersonal communication with street promotions, direct mail-outs, and promotion of doctor or pharmacist recommendations. We expected that women who received mail-out material would talk to each other because they had never previously seen such mail on cancer screening. Compared with mass media and more intensive approaches, direct mail interventions may represent a more promising population-based strategy for promoting cancer screening including mammography [11,12]. Direct mail is a relatively efficient and inexpensive way [13] to reach individuals in their homes, including people not typically exposed to mass media [14,15]. In our study, direct mail had impact on intention toward screening mammography.

As part of this study, we also conducted fifteen group education sessions; these were held in nearly all apartment complexes in the intervention city over 6 months. The education session consists of following parts; OX quizzes to break the myths related to breast cancer and screening mammography, personified story to raise breast cancer awareness, statistics on breast cancer, symptoms of breast cancer, animation that shows how a mammogram is performed, and discussion on fear related to cancer screening. The education evaluation conducted in the pilot test of sampled residents showed that these education sessions improved the participants' knowledge level about both breast cancer and breast cancer screening. However, the limited number of education sessions might be insufficient to generate the critical mass necessary to impact the whole community. Group education by private hospitals (non-campaign) was negatively related to some outcome variables. This finding might be due to negative attitude toward private hospitals that provide the education sessions with intention to promote their hospitals to recruit more patients.

Interestingly, personal stories of cancer patients were inversely associated with intention regarding screening mammography. The literature on cancer-related fear, worry, or anxiety, emotion regulation, and screening behavior is increasing. However, a recent review on this subject reported that the findings of the studies were contradictory [16]. Differences in age, ethnicity, and stages of the cancer screening process might explain these discrepancies.

Several limitations need to be kept in mind when interpreting our data. This study used only 2 cities and, thus, could not guarantee the internal validity expected of randomized multi-city community intervention trials. Therefore, the results cannot be used to estimate true intervention effects or actual screening rates over time. Even though there are curvilinear functions between campaign effectiveness and campaign length, the duration of our campaign could have been too short to sufficiently impact the community. The inclusion of more cities and longer study duration were not possible because our resources were limited. Another limitation of this study is that baseline exposure scores were lower on every activity in the intervention city. A possible explanation is the inclusion of the comparison city in the World Health Organization Korean Healthy City Network in April 2008. A third limitation is that despite some sociodemographic similarities, such as age and education, at both baseline and follow-up in the intervention and comparison cities, the proportion of employed women and women with a history of mammography was different at baseline and follow-up. This suggests some differences in the baseline and follow-up survey populations in the 2 cities. However, those sociodemographic variables were statistically controlled by multivariate logistic regression including CITY, TIME, and CITY by TIME interaction to see whether the campaign activities in the intervention city had an independent influence on the city when we analyzed the effect of each campaign activity. Therefore, any statistically significant campaign activities can be regarded as independent of other variables, including sociodemographic variables.

Because differences in goals, priorities, and values have been frequently found between researchers and communities [17-19], and because such differences could be a major challenge in conducting community campaigns, we tried to harmonize the views of the research team with the community situation and opinions of the community members and public health workers from the study's outset. Although the main non-individual-level theoretical framework employed in most projects has been based on community organization and development models which assume that community participation and coalitions can create a sense of ownership and a synergy of action and outcome that could not otherwise be achieved [20-24], we did not use the community organization model. This was because we thought that the potential of community coalition could be different in terms of the nature of the community issue and its risk. We estimated that the willingness or need for a coalition of community members was not sufficient to use this strategy in our campaign. Another important reason for our decision not to use this strategy was that breast cancer was neither particularly prevalent nor an acutely progressing disease in the intervention city, and few indirect effects were being imposed on the community. Future community intervention trials to improve breast cancer screening should assess the stages of community involvement and coalition before choosing theories to design interventions.

## Conclusions

The data presented here indicate a significant decrease in myths about breast size and the cost of mammography. There was also an increase in intention concerning screening mammography and in the proportion of women at the TTM action stage. These findings suggest that the combination of community outreach and clinic and pharmacy-based in-reach strategies could effectively correct myths related to breast cancer and screening mammography. They could also improve intention toward screening mammography and the stage of adoption for screening mammography for women living in urban Asian communities. Even though evaluation of community-based health interventions involving a comparison city as a control region can be laborious and difficult, such approaches are necessary to garner future support from policy-makers and other key stakeholders.

## Competing interests

The authors declare that they have no competing interests.

## Authors' contributions

KP participated in the conception and design of the study and drafted the manuscript. WHH and SYK participated in the conception and design of the study. EJ participated in the conception and design of the study. MK and HGP participated in design and analysis of the study. All authors gave final approval for the manuscript submitted for publication.

## Pre-publication history

The pre-publication history for this paper can be accessed here:

http://www.biomedcentral.com/1471-2458/11/468/prepub
